# Abnormally activated OPN/integrin αVβ3/FAK signalling is responsible for EGFR-TKI resistance in EGFR mutant non-small-cell lung cancer

**DOI:** 10.1186/s13045-020-01009-7

**Published:** 2020-12-07

**Authors:** Yulong Fu, Yang Zhang, Zhe Lei, Ting Liu, Tingting Cai, Anqi Wang, Wenwen Du, Yuanyuan Zeng, Jianjie Zhu, Zeyi Liu, Jian-an Huang

**Affiliations:** 1 Department of Pulmonary and Critical Care Medicine, The First Affiliated Hospital of Soochow University, Suzhou, 215006 People’s Republic of China; 2 Institute of Respiratory Diseases, Soochow University, Suzhou, 215006 People’s Republic of China; 3 Department of Genetics, School of Biology and Basic Medical Sciences, Medical College of Soochow University, Suzhou, 215123 People’s Republic of China; 4 Suzhou Key Laboratory for Respiratory Diseases, Suzhou, 215006 People’s Republic of China

**Keywords:** Osteopontin, Integrin, EGFR-TKI, Resistance, NSCLC

## Abstract

**Background:**

Acquired epidermal growth factor receptor tyrosine kinase inhibitor (EGFR-TKI) resistance limits the long-term clinical efficacy of tyrosine kinase-targeting drugs. Although most of the mechanisms of acquired EGFR-TKI resistance have been revealed, the mechanism of ~ 15% of cases has not yet been elucidated.

**Methods:**

Cell viability was analysed using the Cell Counting Kit-8 (CCK-8) assay. Proteome profiler array analysis was performed to find proteins contributing to acquired EGFR-TKI resistance. Secreted OPN was detected by ELISA. Immunohistochemical analysis was conducted to detect expression of integrin αV in NSCLC tissue. The effect of VS-6063 on apoptosis and proliferation of PC9 gefitinib-resistant cells was detected by fluorescence-activated cell sorting (FACS) and clonogenic assays. A mouse xenograft model was used to assess the effect of VS-6063 on the sensitivity of PC9 gefitinib-resistant cells to gefitinib.

**Results:**

OPN was overexpressed in acquired EGFR-TKI-resistant NSCLCs. Secreted OPN contributed to acquired EGFR-TKI resistance by activating the integrin αVβ3/FAK pathway. Inhibition of FAK signalling increased sensitivity to EGFR-TKIs in PC9 gefitinib-resistant cells both in vitro and in vivo.

**Conclusions:**

OPN contributes to acquired EGFR-TKI resistance by up-regulating expression of integrin αVβ3, which activates the downstream FAK/AKT and ERK signalling pathways to promote cell proliferation in NSCLC.

## Background

Epidermal growth factor receptor tyrosine kinase inhibitors (EGFR-TKIs), including gefitinib, erlotinib, afatinib, and dacomitinib, are effective as first-line treatment for advanced non-small-cell lung cancer (NSCLC) harbouring activating *EGFR* mutations (e.g., deletions in exon 19 and the exon 21 L858R mutation) [1–5]. *EGFR* T790M mutation emerges following EGFR-TKI therapy, which accounts for 55% of acquired resistance to first- and second-generation EGFR-TKIs [6, 7]. In addition, the molecular alternations that lead to EGFR-TKI resistance include bypass pathway activation [e.g., MET amplification (MET-amp), HER2 amplification (HER2-amp)] and downstream signalling pathways activation (e.g., PI3K and BRAF mutations) [6, 8, 9]. Histological transformations [e.g., small cell and epithelial-mesenchymal transition (EMT)] are also involved in TKI resistance [10, 11]. However, the mechanism remains unknown in ~ 15% of patients with acquired resistance to EGFR-TKIs.

Osteopontin (OPN) is a secretory extracellular matrix glycosylated phosphoprotein that was first identified in bone tissue as a major sialoprotein in modulating bone formation and remodelling [12]. It is a member of the small integrin-binding ligand N-linked glycoproteins, a family of five integrin binding glycophosphoproteins, including bone sialoprotein, dentin matrix protein 1, dentin sialophosphoprotein, and matrix extracellular phosphoglycoprotein [13]. OPN is an extracellular matrix (ECM) ligand for integrins and a likely candidate to promote angiogenesis in the uterus and placenta. OPN is highly expressed in osteoblasts and osteoclasts. OPN also plays an important role in biomineralization [14], and it also contributes to various metastasis-associated mechanisms, including proliferation, survival, adhesion, migration, invasion, and angiogenesis [15–17]. Moreover, OPN has been demonstrated to play a role in the metastasis of NSCLC. OPN is up-regulated in NSCLC and even more in cells with strong potential and capacity of metastasis and invasion [18, 19], which can be attenuated by its deletion [20]. Up-regulation of OPN is proposed to be associated with stages, severities, lymph node metastasis, poor prognosis, and high recurrence [21–23]. However, it is still unclear whether OPN is responsible for acquired resistance to EGFR-TKIs.

This study aims to identify whether expression of OPN correlates with acquired resistance as well as the exact signalling pathways involved in OPN-mediated acquired resistance to EGFR-TKIs.

## Materials and methods

### Cell culture and reagents

PC9, HCC827 and H1975 cell lines were purchased from the Cell Bank of Type Culture Collection of the Chinese Academy of Sciences (Shanghai, China). Caicun Zhou at Tongji University School of Medicine provided PC9GR cells. HCC827GR cells were derived from HCC827 cells by exposure to gefitinib, as previously described [24]. PC9 and PC9GR cells were routinely cultured in RPMI-1640 medium supplemented with 10% foetal bovine serum (Gibco, Carlsbad, CA, USA). HCC827, HCC827GR, and H1975 cells were cultured in RPMI-1640 medium supplemented with 10% heat-inactivated FBS (Gibco). Cells were grown in a humidified incubator with 5% CO_2_ at 37 °C.

Gefitinib was supplied by AstraZeneca (London, UK). Osimertinib and the FAK inhibitor VS-6063 were purchased from Selleck Chemicals (Houston, TX, USA).

### Viability and proliferation assays

Cells were plated in each well of a 96-well plate at a density of 3000 cells per well, grown overnight and then treated with varying drug concentrations for 72 h. The Cell Counting Kit-8 (CCK-8) assay kit (Boster, Wuhan, China) was used according to the manufacturer’s instructions to assess cell viability. Fluorescence at 630 nm and 450 nm was measured using a microplate reader after 1–2 h (Thermo, Waltham, MA, USA).

### Proteome profiler array analysis

The protein profile was analysed using a human soluble receptor array kit, non-haematopoietic panel-ARY012 (R&D Inc. Minneapolis, MN, USA) according to the manufacturer’s protocols. PC9, HCC827, PC9GR and HCC827R cells were lysed with lysis buffer mixed with proteinase cocktail inhibitor (Roche, Branford, CT, USA). Cell lysates were pipetted up and down for resuspension on ice for 30 min and then centrifuged at 14,000 × g at 4 °C for 5 min. The protein lysates were collected, and the concentrations were determined by the bicinchoninic acid assay (BCA assay). The protein lysates (100–300 μg per membrane) were incubated overnight with nitrocellulose membranes containing 62 soluble receptors. The membranes were subsequently incubated first with a specific cocktail of biotinylated detection antibodies and later with the streptavidin–horseradish peroxidase solution. Signals were detected by using a chemifluorescence detection system (Bio-Rad, Hercules, CA, USA) according to the manufacturer’s protocol. The relative density of specific protein expression was determined using Quantity One software.

### Kinase and western blot assays

We used Human Phospho-Kinase Array Kit-ARY003B (R&D) for the human receptor tyrosine kinase (RTK) assay. Cells were seeded into 6-well plates at a concentration of 3 × 10^5^ cells/well. After 24 h, the cells were harvested and lysed in RIPA buffer (Cell Signalling Technology, Danvers, MA, USA) containing a protease and phosphatase inhibitor cocktail (Sigma-Aldrich, Louis, MO, USA). The protein lysates were incubated with the array membrane, and the protein signal was visualized using chemifluorescent detection (Bio-Rad) according to the manufacturer’s protocol. The relative density of specific protein expression was determined using Quantity One software.

Antibodies against the following were obtained from Cell Signalling Technology: p-EGFR Y1068 (#2234), ERK (#9102), p-ERK T202/Y204 (#9101), AKT (#9272), p-AKT S473(#4060), FAK (#3285), p-FAK Y397 (#8556), SRC (#2108), p-SRC Y416 (#2101), and PARP (#9542). The β-actin antibody (CW0096M), GAPDH antibody (CW0100M), horseradish peroxidase (HRP)-conjugated anti-mouse antibody (CW0102), and HRP-conjugated anti-rabbit antibody (CW0103) were purchased from CoWin Biosciences (Beijing, China). Anti-EGFR (C-2), OPN (LFMb-14), -integrin αV (P2W7), and -integrin β3 (B-7) antibodies were purchased from Santa Cruz Biotechnology. The anti-integrin β1 (AF5379) antibody was purchased from Affinity Biotechnology. For immunoblotting, cells were harvested, washed in PBS, and lysed in RIPA buffer [50 mmol/L Tris–HCl (pH 8.0), 150 mmol/L sodium chloride, 5 mmol/L magnesium chloride, 1% Triton X-100; 0.5% sodium deoxycholate, 0.1% SDS, 40 mmol/L sodium fluoride, 1 mmol/L sodium orthovanadate, and complete protease inhibitors (Selleck Chemicals, Houston, TX, USA)]. Western Lightning ECL reagent (Thermo) was used for signal detection.

### siRNA experiments

Transfection was carried out using Lipofectamine 2000 transfection reagent (Invitrogen, Carlsbad, NM, USA) according to the manufacturer's protocol. The target siRNA sequences used are listed in Additional file 1: Table S1. siRNAs were used at a concentration of 10 nmol/L (GenePharma, Shanghai, China). The efficacy of transfection was verified by qRT-PCR.

### Flow cytometry analysis

Cells were seeded in 6-well plates at a density of 5 × 10^4^ cells per well and treated with drugs at different concentrations or DMSO as a negative control. We analysed apoptosis and the cell cycle status of the cells by using Annexin V-FITC and propidium iodide (PI) (R&D) staining according to the manufacturer’s protocol.

### ELISA

The secreted OPN (sOPN) was measured using ELISA (MultiSciences, Hangzhou, China). Briefly, 50 μL of diluent was added to each well of the microplate. Then, another 50 μL standard, control, or sample was added to each well and incubated for 2 h at room temperature. After washing four times, the conjugate reagent was added to each well and incubated for 2 h at room temperature. After washing four times, 100 μL substrate solution was added to each well, and the reaction was stopped after 30 min by adding the stop solution. Absorbance at 450 nm was measured using a spectrophotometer (Thermo). PC9GR cells were exposed to medium containing 1% FBS for 12 h and then treated with the following inhibitors separately: the autophagy inhibitor 3-methyladenine (1 mM, Selleck Chemicals), the protein transport inhibitor brefeldin A (10 ng/ml, APExBIO, Houston, TX, USA), and the exosome secretion inhibitor 5-(*N*, *N*-dimethyl)- amiloride DMA (50 nM, APExBIO). The supernatant was collected from the cultured medium 24 h later.

### RNA extraction and quantitative real-time PCR analysis

Total RNA was extracted from cells using TRIzol reagent (Invitrogen) and reverse transcribed to cDNA using reverse transcription reagents (Takara Bio, Shiga, Japan) according to the manufacturer's protocol. The primer sequences used for reverse transcription quantitative polymerase chain reaction (qRT‐PCR) are listed in Additional file 1: Table S1. Quantitative RT-PCR was performed using SYBR Premix ExTaq™ (Takara) with an ABI StepOnePlus Real-Time PCR system (Applied Biosystems, Foster City, CA) according to the operator's manual. The expression values of genes were normalized to the internal control GAPDH.

### Human tissue and IHC

Seven NSCLC tissue samples were collected from patients between 2017 and 2018 at the Respiratory Department of the First Affiliated Hospital of Soochow University. All participants provided written informed consent at the time of recruitment. All cases had clinically and pathologically confirmed diagnoses of NSCLC in accordance with the Revised International System for Staging Lung Cancer.

Immunohistochemical (IHC) analysis was conducted in our previous study. Briefly, sections were incubated with anti-ITGαV (EPR16800, 1:200 dilution; Abcam) overnight at 4 °C and then with biotinylated secondary antibodies. The reactions were developed using DAB Kit (BD Bioscience, San Jose, CA, USA), and the sections were counterstained with haematoxylin. The staining area was scored using the following scale: 0, 0–10% of tissue stained positive; 1, 10–20% stained positive; 2, 20–40% stained positive; 3, 40–70% stained positive; and 4, > 70% positive cells. The IHC score was generated from three different areas of the slides, and the average score was calculated for each sample.

### Immunofluorescence staining

Cultured cells were fixed with 4% paraformaldehyde for 15 min at room temperature, permeabilized with Triton (0.1% in TBS) for 30 min and blocked with 5% BSA in PBS for 1 h at room temperature. The cells were then incubated overnight at 4 °C with anti-ITGαV antibodies (EPR16800, 1:200 dilution; Abcam) followed by Alexa Fluor 488-conjugated anti-rabbit IgG (Beyotime, Shanghai, China) for 90 min. Finally, the samples were incubated in DAPI for 10 min (Life Technologies) for nuclear counterstaining. Images were acquired using a Leica SP8 confocal microscope with optimal settings for the fluorescent markers used.

### Mouse xenograft models and establishment of EGFR-TKI-resistant lung cancer tumours in vivo

Male athymic BALB/c nude mice were purchased from the Experimental Animal Center of Soochow University and bred under pathogen-free conditions. All experimental procedures were reviewed and approved in accordance with the guidelines for the care and use of laboratory animals, and informed written consent was obtained from Soochow University. To establish mouse xenograft models, the same amount of the indicated tumour cells was injected subcutaneously into both flanks of each mouse. Tumour volumes (mm^3^) were calculated as length × width^2^/2. When tumours reached ~ 200 mm^3^, gefitinib and VS-6063 were given by gavage at 12.5 mg/kg and 25 mg/kg daily, respectively, until the mice were sacrificed.

### Statistical analysis

All results are presented as the mean standard deviation (SD). Two-way ANOVA was used to calculate the difference in IC50 for EGFR-TKIs among cells with various treatments. Statistical comparisons were determined with Student's *t* test, and *P* < 0.05 was regarded as significant. All statistical analyses were performed with GraphPad Prism 7.0 (GraphPad, San Diego, CA) and SPSS 17.0 software (SPSS, Chicago, IL).

## Results

### OPN is involved in acquired EGFR-TKI resistance in NSCLC

**Fig. 1:**
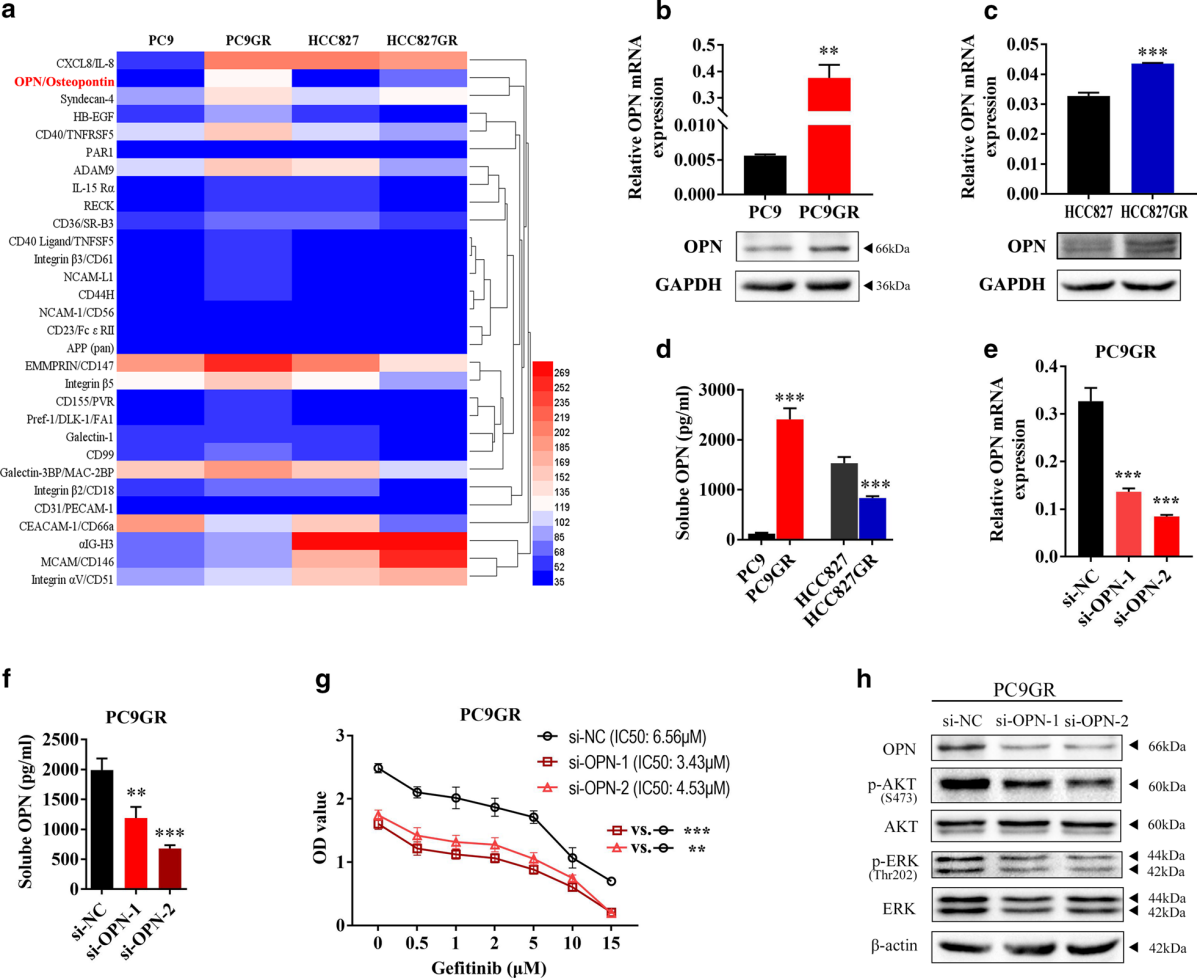
OPN is involved in acquired EGFR-TKI resistance in NSCLC. **a** Expression of soluble receptors and related proteins was examined by proteome profiler array analysis in HCC827, PC9, HCC827GR and PC9 GR cells. **b**, **c** The expression level of OPN was determined by quantitative real-time PCR and western blot analysis. **d** Expression of secreted OPN (sOPN) was verified by ELISA in HCC827GR and PC9GR cells. **e**, **f** qRT-PCR and ELISA were used to detect expression of OPN in PC9GR cells after transfection with si-OPN. **g** The sensitivity of PC9GR cells transfected with si-OPN or si-NC to gefitinib was determined by CCK-8 assays. **h** Western blot analysis was used to detect expression of p-AKT and p-ERK in HCC827GR cells transfected with si-OPN or si-NC (**P < 0.01; ***P < 0.001)

To determine which specific protein contributes to acquired EGFR-TKI resistance in NSCLC, two EGFR mutant NSCLC cell lines, PC9 and HCC827 [25], were exposed to gefitinib at gradually increasing concentrations for more than 6 months to establish gefitinib-resistant (GR) cell lines (PC9GR and HCC827GR), as previously reported [24]. PC9GR and HCC827GR cells were highly insensitive to gefitinib compared to their respective parental cells (Additional file 2: Fig S1). Our previous work showed that the PC9GR cell line harbours EGFR 19del and T790M mutations; the HCC827GR cell line harbours the EGFR 19del mutation and overexpresses FGFR1 and AXL [26, 27]. To investigate whether there are other mechanisms responsible for acquired resistance to EGFR-TKIs in addition to variations such as the T790M mutation, MET amplification and overexpression of FGFR1 and AXL, we examined expression of soluble receptors and related proteins in PC9GR and HCC827GR cells by proteome profiler array analysis. We found that OPN was the only protein up-regulated simultaneously in the two EGFR TKI-resistant cell lines (fold change > 1.5) (Fig. 1a, Additional file 3: Table S2 and Additional file 4: Fig S2). Then, OPN up-regulation in PC9GR and HCC827GR cells was verified by qRT‐PCR and western blot assays (Fig. 1b, c). As OPN is considered to perform multiple functions as a canonical secretory protein [16], we detected expression of secreted OPN (sOPN) by ELISA. sOPN expression was apparently higher in PC9GR cells than in PC9 cells. In contrast, HCC827GR cells expressed less sOPN than HCC827 cells (Fig. 1d). In addition, we found OPN could be secreted via Golgi apparatus, secretory autophagy and exosome in PC9GR cells (Additional file 5: Fig. S3). These results suggest that OPN is more associated with EGFR-TKI resistance in PC9 cells. Therefore, further explorations were mainly performed using PC9 and PC9GR cells. To examine whether OPN is positively associated with acquired resistance to EGFR-TKIs, OPN mRNA and protein were significantly silenced by siRNA transient transfection in PC9GR cells (Fig. 1e). Secretory OPN was also down-regulated after si-OPN transfection (Fig. 1f). A cell viability (CCK8) assay demonstrated that silencing OPN significantly increased the gefitinib sensitivity of PC9GR cells (Fig. 1g). Furthermore, silencing OPN blocked the phosphorylation of AKT and ERK (Fig. 1h). Interestingly, OPN secretion was increased not only in gefitinib-resistant cells (PC9GR) but also in osimertinib-resistant cells (PC9GR/OR) (Additional file 6: Fig. S4A and B). These results suggest that OPN promotes acquired gefitinib resistance in NSCLC cells.

### Integrin αVβ3 contributes to acquired EGFR-TKI resistance in NSCLC

**Fig. 2:**
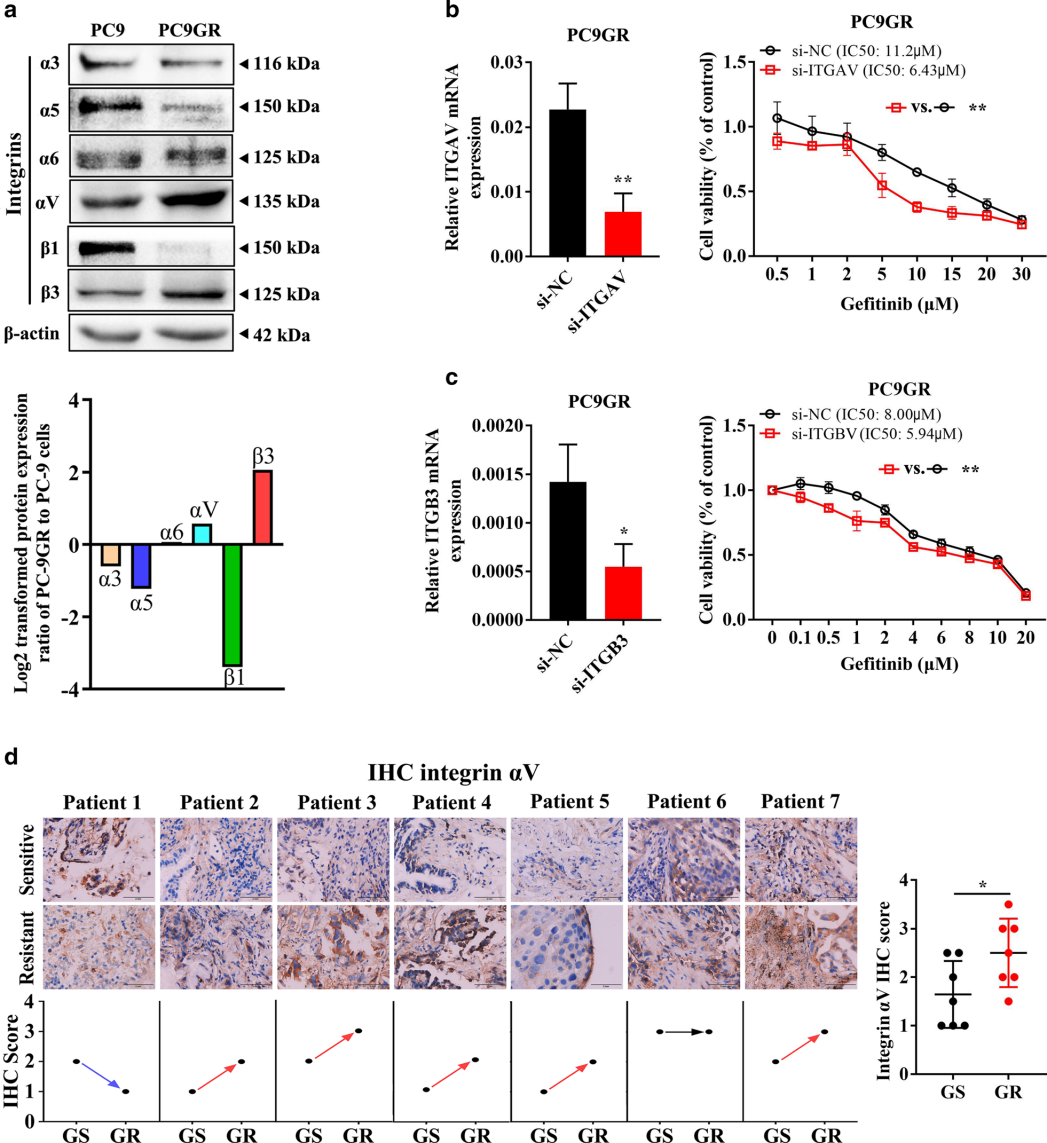
Integrin αVβ3 contributes to acquired EGFR-TKI resistance in NSCLC. **a** Expression of integrins was determined by western blot analysis. **b** The sensitivity of PC9GR cells transfected with si-ITGAV or si-NC to gefitinib was determined by CCK-8 assays. **c** The sensitivity of PC9GR cells transfected with si-ITGB3 or si-NC to gefitinib was determined by CCK-8 assays. **d** Integrin αV expression was detected in seven tumour tissues from lung adenocarcinoma patients before and after EGFR-TKI resistance by IHC (*P < 0.05; **P < 0.01)

Given that OPNs are reported to bind to multiple integrins through Arg-Gly-Asp (RGD)-mediated integrin recognition sequences or alternative integrin recognition sequences to mediate cell–cell and cell-ECM interactions [28, 29], we hypothesize that integrins are involved in OPN-mediated acquired resistance to EGFR-TKIs. As shown in Fig. 2a, integrins αV and β3 were remarkably overexpressed in PC9GR cells compared to PC9 cells, which is consistent with the results of the proteome profiler array analysis (Additional file 3: Table S2 and Additional file 4: Fig S2). RNA data from the database TCGA showed that mRNA expression of ITGAV and ITGB3 correlated positively with SPP1 expression in lung adenocarcinoma and squamous patients (Additional file 7: Fig. S5). Furthermore, the sensitivity of PC9GR cells to gefitinib was increased with knockdown of integrin αV (Fig. 2b) or integrin β3 (Fig. 2c). Integrin αV expression was detected in 7 tumour tissues from lung adenocarcinoma patients before and after EGFR-TKI resistance acquisition (Additional file 8: Table S3). As shown in Fig. 2d, expression of integrin αV was increased in five patients with EGFR-TKI resistance (5/7 = 71.43%) but was not changed in patient 6 with MET amplification. These data demonstrate that integrin αVβ3 contributes to acquired EGFR-TKI resistance in NSCLC.

### Activation of the FAK signalling pathway mediates acquired EGFR-TKI resistance in NSCLC

**Fig. 3:**
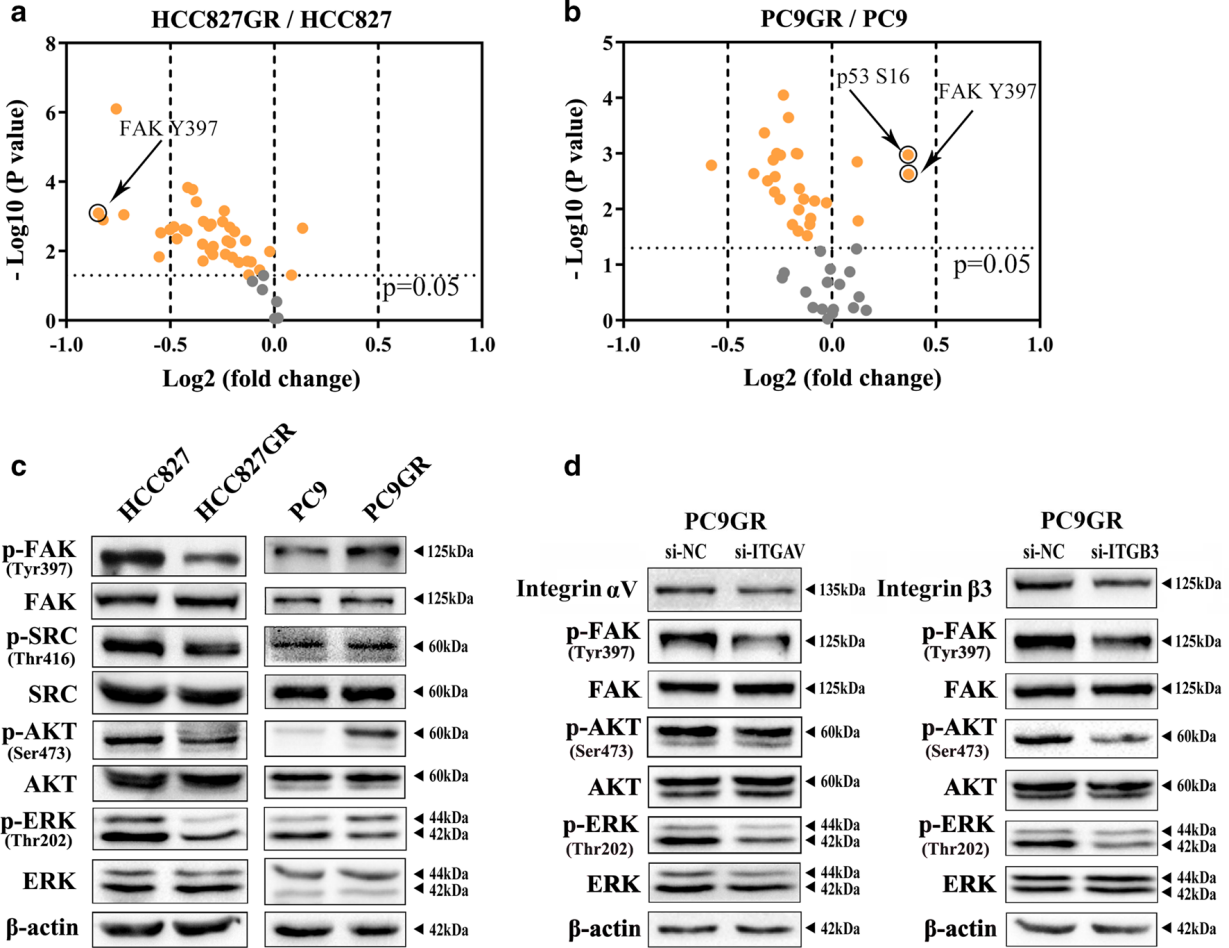
Activation of the FAK signalling pathway mediates acquired EGFR-TKI resistance in NSCLC. **a**, **b** Activity of protein kinases in PC9, PC9GR, HCC827, and HCC827GR cells using kinome-wide array analysis. **c** Western blotting was used to analyse expression of p-FAK, p-SRC, p-AKT, and p-ERK in HCC827, HCC827GR, PC9, and PC9GR cells. **d** Expression of p-FAK, p-AKT and p-ERK in PC9GR cells transfected with si-ITGAV or si-ITGB3 was detected by western blot analysis

To identify kinases that are related to EGFR-TKI in EGFR mutant NSCLC, we compared the difference in activity of protein kinases between PC9 and PC9GR cells and between HCC827 and HCC827GR cells using kinome-wide array analysis (Additional file 9: Table S4 and Additional file 10: Fig S6). As shown in Fig. 3a, b, phosphorylated FAK (Tyr397) was activated in PC9GR cells in comparison with PC9 cells (Fig. 3a) but was reduced in HCC827GR cells compared with HCC827 cells (Fig. 3b). These results were further verified by western blot analysis (Fig. 3c). Consistent with FAK, its downstream factors, including SRC, AKT and ERK, were phosphorylated and activated in PC9GR cells. In addition, silencing integrins αV and β3 reduced the phosphorylation of FAK and its downstream factors in PC9GR cells (Fig. 3d). These results suggest that activation of the FAK signalling pathway mediates acquired EGFR-TKI resistance in NSCLC.

### OPN enhances gefitinib-activated FAK signalling by up-regulating integrin αVβ3 expression

**Fig. 4:**
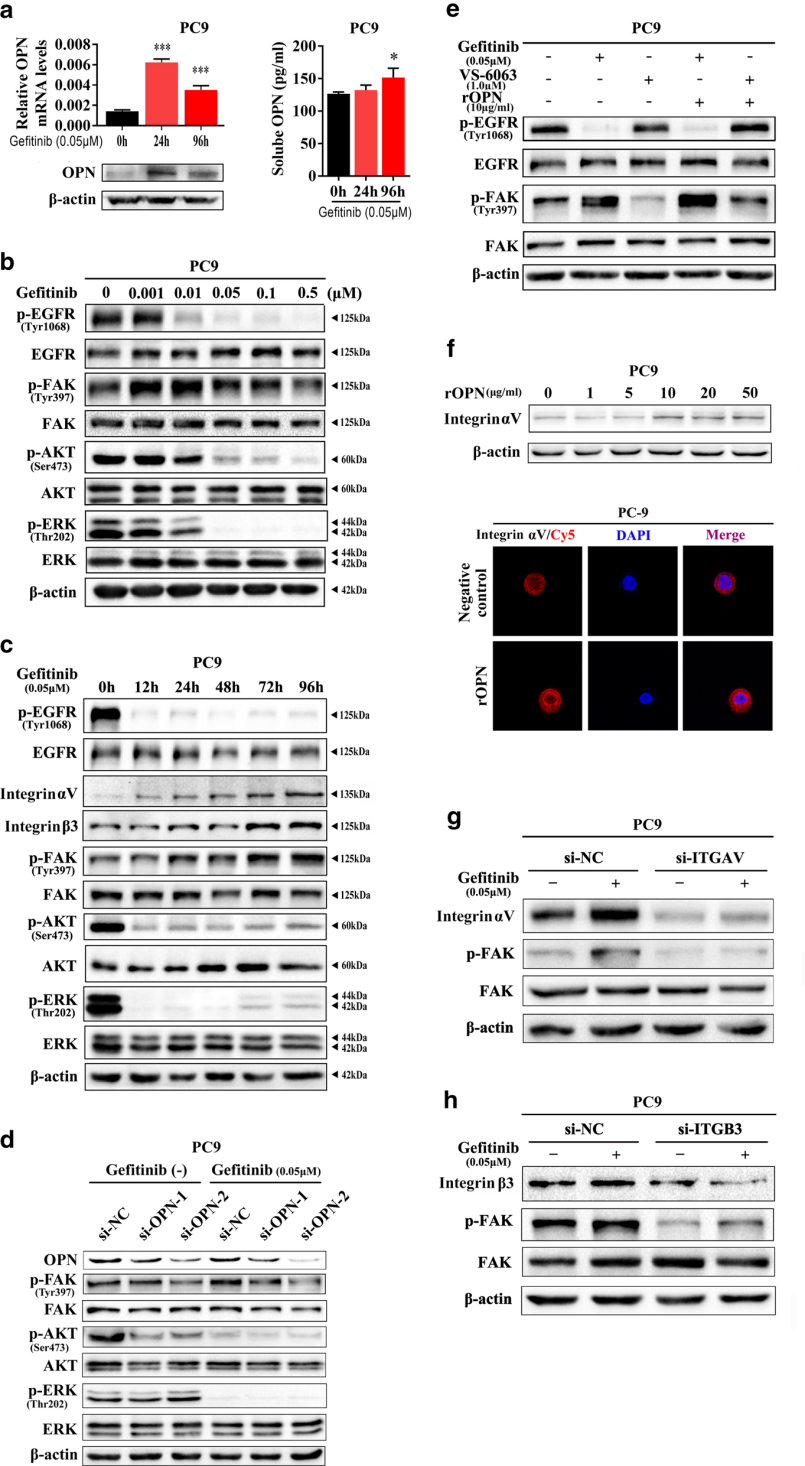
OPN enhances gefitinib-activated FAK signalling by up-regulating integrin αVβ3 expression. **a** Under gefitinib treatment, OPN expression was detected by qRT-PCR, western blot analysis and ELISA. **b**, **c** Western blot analysis was used to detect the effect of gefitinib treatment on p-EGFR, p-FAK, p-AKT and p-ERK in PC9 cells at different concentrations and times. **d** Under gefitinib treatment, p-FAK, p-AKT and p-ERK levels in PC9 cells transfected with si-OPN or si-NC were detected by western blot analysis. **e** The effect of single-drug or combined treatment of rOPN and VS-6063 on p-FAK in PC9 cells. **f** Expression of integrin αV was detected by western blot and immunofluorescence after PC9 cells were treated with different concentrations of rOPN. **g**, **h** Under gefitinib treatment, p-FAK in PC9 cells transfected with si-ITGAV or si-ITGB3 was detected by western blot analysis (*P < 0.05; ***P < 0.001)

Because we found that OPN plays a role in increasing gefitinib resistance, it is natural to assume that integrins and FAK signalling are involved in this process. Expression of OPN (Fig. 4a, left) and secretion of sOPN (Fig. 4b, right) was up-regulated in PC9 cells treated with gefitinib. As illustrated in Fig. 4b, phosphorylation of EGFR and downstream AKT and ERK activities were significantly suppressed by gefitinib treatment at concentrations greater than 0.05 μM. In contrast, FAK phosphorylation was markedly enhanced in PC9 cells treated with 0.01 μM gefitinib (Fig. 4b). Moreover, upon 0.05 μM gefitinib stimulation, levels of p-FAK, as well as of integrins αV and β3, were gradually up-regulated with treatment time (Fig. 4c). Moreover, expression of p-AKT and p-ERK, which were inhibited by gefitinib-blocked p-EGFR, was partially restored after 72 h of gefitinib stimulation (Fig. 4c). As expected, we found that knockdown of OPN significantly prevented FAK phosphorylation as well as gefitinib-induced FAK phosphorylation in PC9 cells (Fig. 4d). FAK phosphorylation was also significantly activated by treatment with recombinant human OPN (rOPN), and in turn, this process was blocked by VS-6063, a specific inhibitor of FAK activity in PC9 cells (Fig. 4e). Furthermore, integrin αV was up-regulated by recombinant human OPN (rOPN) treatment, as determined by western blot (upper) and immunofluorescence (lower) assays (Fig. 4f). Finally, silencing integrin αV (Fig. 4g) or β3 (Fig. 4h) suppressed gefitinib-induced FAK phosphorylation, which mirrored the results of OPN knockdown. Given that OPN interacts mainly with various integrin αVs (particularly αVβ1 and αVβ3) [30, 31], it is necessary to determine whether integrin β1 is also involved in FAK signalling. We found that knockdown of integrinβ1 did not affect FAK activity after stimulation with gefitinib in PC9GR cells (Additional file 11: Fig. S7). These results indicate that OPN activates gefitinib-induced FAK signalling by up-regulating integrin αVβ3 expression in NSCLC.

### FAK signalling pathway contributes to acquired resistance to osimertinib in NSCLC

**Fig. 5:**
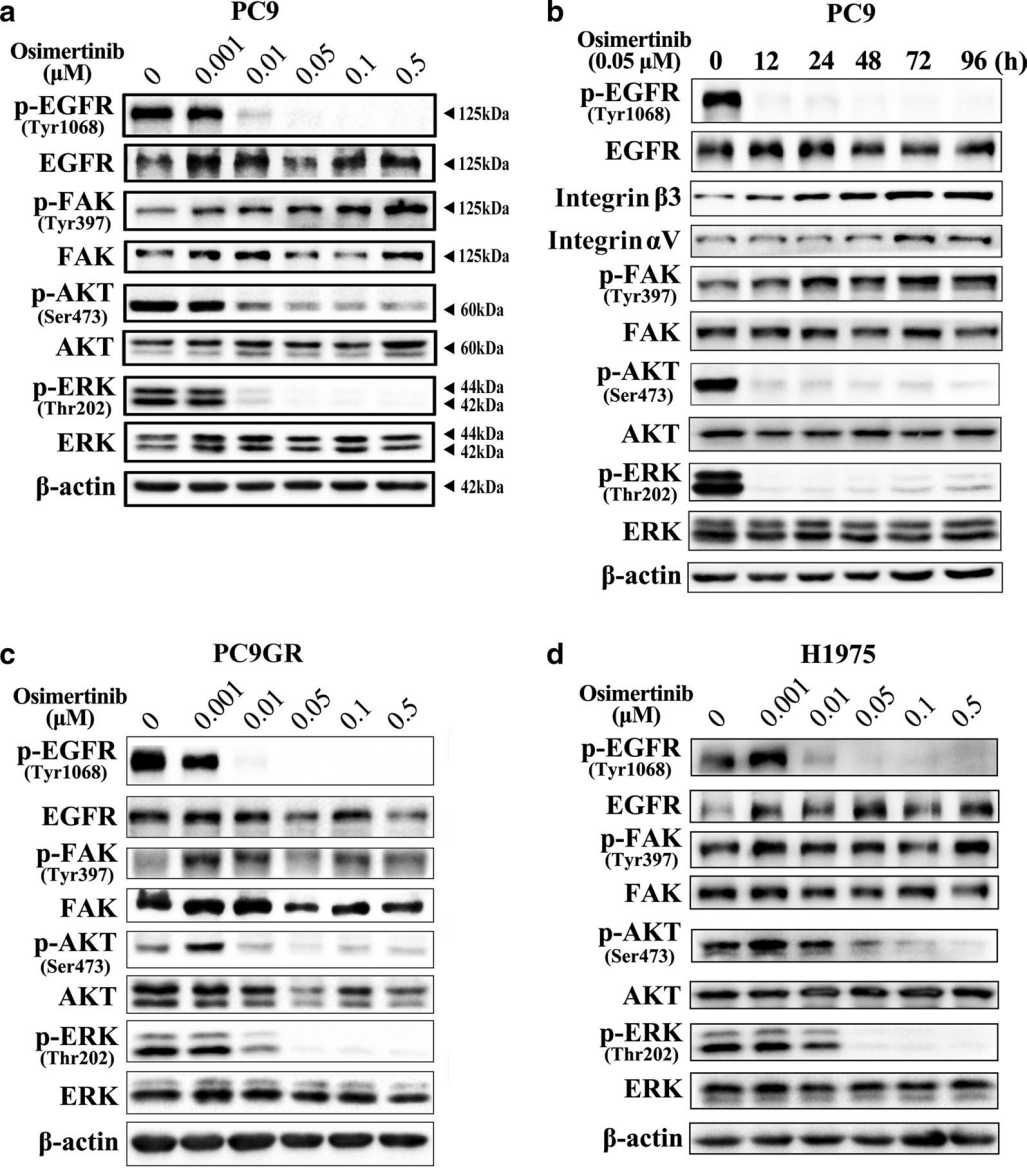
The FAK signalling pathway contributes to acquired resistance to osimertinib in NSCLC. **a**, **b** Western blot analysis was used to detect the effect of osimertinib treatment on p-FAK, p-AKT and p-ERK in PC9 cells at different concentrations and times. **c**, **d** Western blot analysis was used to detect the effect of osimertinib treatment on p-EGFR, p-FAK, p-AKT, and p-ERK in PC9GR and H1975 cells at different concentrations

To investigate whether the FAK signalling pathway contributes to acquired resistance to different EGFR-TKIs, we examined expression of p-FAK in PC9 cells treated with osimertinib at various concentrations and times. Similar to the results obtained with gefitinib treatment, p-FAK expression increased with increasing osimertinib concentration and time (Fig. 5a, b). Moreover, expression of integrin αvβ3 gradually increased with the extension of 0.05 μM osimertinib treatment time (Fig. 5b). Given that osimertinib is an oral, irreversible EGFR-TKI that is selective for both EGFR and T790M resistance mutations [32], we also evaluated p-FAK in T790M mutant cells (PC9GR and H1975) treated with osimertinib [25, 27]. As shown in Fig. 5c, d, p-FAK levels were elevated in PC9GR and H1975 cells treated with different concentrations of osimertinib. These results suggest that the FAK signalling pathway contributes to acquired resistance to osimertinib in NSCLC.

### Inhibition of the FAK signalling pathway increases sensitivity to EGFR-TKI in PC9 cells

**Fig. 6:**
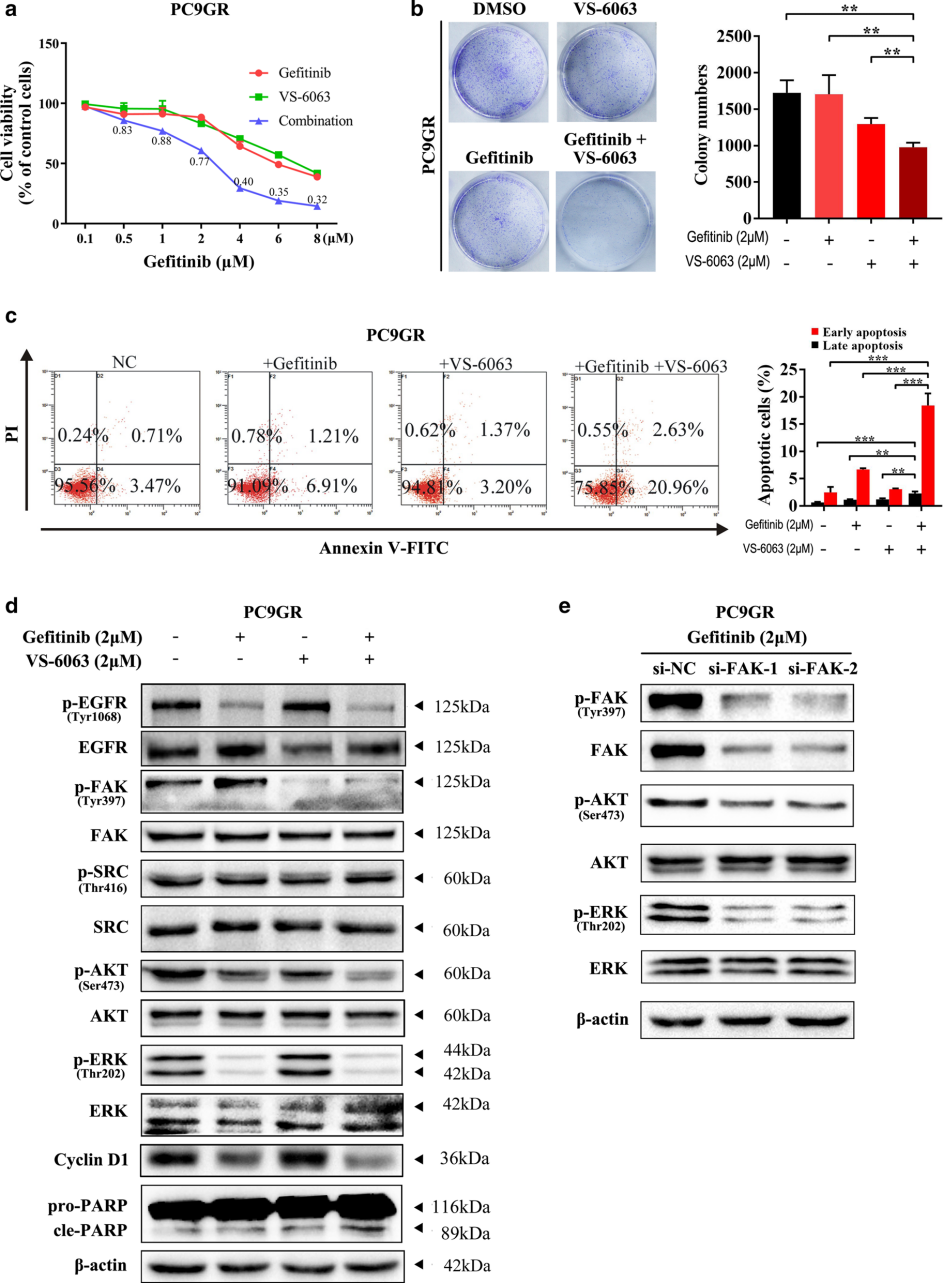
Inhibition of the FAK signalling pathway increases sensitivity to EGFR-TKIs in PC9 cells. **a** The sensitivity of PC9GR cells treated with VS-6063 to gefitinib was determined by CCK-8 assays. **b** The effect of gefitinib combined with VS-6063 treatment on the proliferation of PC9GR cells. **c** Fluorescence-activated cell sorting (FACS) analysis was performed to investigate the effects of gefitinib combined with VS-6063 treatment on apoptosis in PC9GR cells. **d** Western blot analysis was used to detect the effect of gefitinib combined with VS-6063 treatment on CyclinD1 and PARP in PC9GR cells. **e** Under gefitinib treatment, p-FAK, p-AKT and p-ERK levels in PC9 cells transfected with si-FAK or si-NC were detected by western blot analysis (**P < 0.01; ***P < 0.001)

**Fig. 7:**
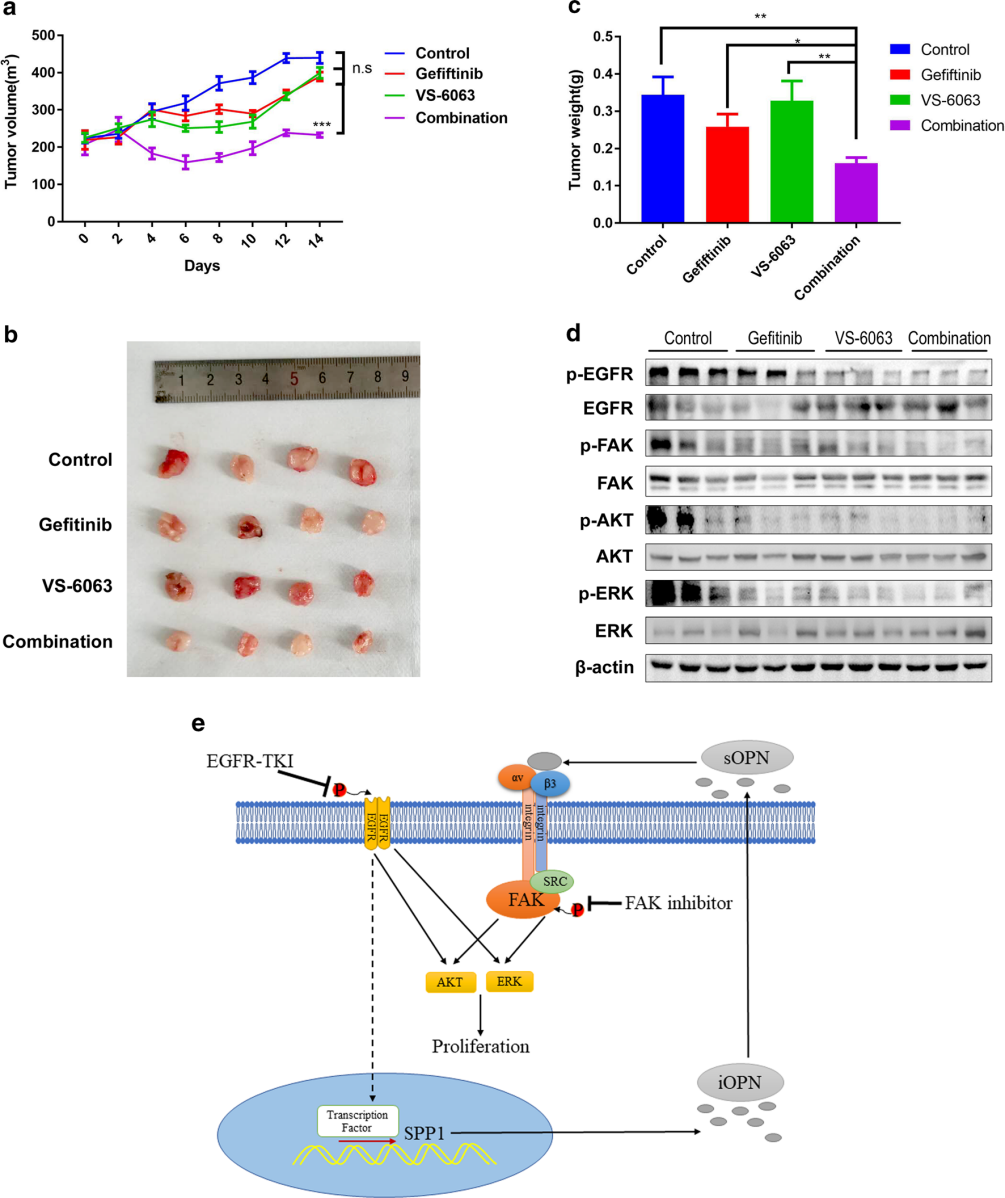
VS-6063 increases the sensitivity of PC9GR cells to gefitinib in vivo. **a** Athymic nude mice with PC9GR tumours were treated with gefitinib (12.5 mg/kg) or gefitinib (12.5 mg/kg) plus VS-6063 (25 mg/kg) for 2 weeks followed by treatment cessation. The tumour volume was measured at the indicated time intervals and calculated. **b**, **c** At the end of treatment, the tumours were excised, photographed as indicated and weighed. **d** Western blotting was used to analyse expression of p-EGFR, p-FAK, p-AKT and p-ERK in tumour tissues of different treatment groups. **e** Schematic diagram depicting the roles of OPN in acquired EGFR-TKI resistance in NSCLC by activating the integrin αVβ3/FAK signalling pathway (*P < 0.05; **P < 0.01; ***P < 0.001)

To verify whether the FAK signalling pathway is involved in acquired resistance to EGFR-TKIs, we explored the gefitinib sensitivity of PC9GR after treatment with a p-FAK inhibitor (VS-6063). As shown in Fig. 6a, when PC9GR cells were treated with VS-6063, their sensitivity to gefitinib was significantly increased. A cell proliferation assay indicated that gefitinib in combination with VS-6063 significantly inhibited PC9GR cell growth compared with each single agent (Fig. 6b). Moreover, flow cytometry results indicated that gefitinib in combination with VS-6063 significantly increased PC9GR cell apoptosis compared with each single agent (Fig. 6c). Then, we detected Cyclin D1 and cleaved PARP in PC9 GR cells treated with VS-6063 in the presence of gefitinib by western blot assay. When PC9GR cells were treated with gefitinib and VS-6063, we found that compared to single-agent treatment, Cyclin D1 and cleaved PARP levels were increased, accompanied by decreased phosphorylation of AKT and ERK (Fig. 6d). In the presence of gefitinib, silencing FAK also reduced phosphorylation of AKT and ERK (Fig. 6e). To verify our results, we assessed the effect of gefitinib in combination with VS-6063 in a mouse xenograft model. As shown in Fig. 7a–c, the effect of gefitinib combined with VS-6063 on inhibiting the growth of PC9GR cells was stronger than that of each single drug. Furthermore, we verified the inhibition of AKT and ERK phosphorylation by combination therapy by western blotting (Fig. 7d). The results indicated that inhibition of the FAK signalling pathway increased sensitivity to EGFR-TKIs in NSCLC. Taken together, our findings reveal that OPN contributes to acquired EGFR-TKI resistance by up-regulating expression of integrin αVβ3, which activates the downstream FAK/AKT and ERK signalling pathways to promote cell proliferation in NSCLC (Fig. 7i).

## Discussion

Acquired EGFR-TKI resistance limits the long-term clinical efficacy of these drugs. Although most of the mechanisms of acquired EGFR-TKI resistance have been revealed, the mechanism of ~ 15% of cases has not yet been elucidated. In this study, we report that OPN contributes to acquired EGFR-TKI resistance by up-regulating expression of integrins αv and β3, which activates the downstream FAK/AKT and ERK signalling pathways to promote cell proliferation in NSCLC. These results provide theoretical bases for novel alternatives and treatment strategies for patients with EGFR-TKI-acquired resistant NSCLC.

To identify novel mechanisms contributing to EGFR-TKI acquired resistance in NSCLC, we performed proteome profiler array analysis in gefitinib-sensitive parent cell lines (PC9 and HCC827) and gefitinib-resistant cell lines (PC9GR and HCC827GR). A total of 119 soluble receptors and related proteins were detected. Interestingly, OPN was most significantly increased in both resistant cell lines, indicating that OPN plays an important role in EGFR-TKI acquired resistance in NSCLC. However, secretory OPN was only increased in PC9GR cells and not in HCC827GR cells (Fig. 1b). This might be because OPN secretion in HCC827GR cells is regulated, leading to a different mechanism of EGFR-TKI resistance in HCC827GR cells from that in PC9GR cells. OPN is a secretory extracellular matrix glycosylated phosphoprotein [12]. High expression of SPP1 was reported to be involved in tumour invasion, progression, and metastasis in multiple cancers, including breast, ovarian, and colon cancer [33–35]. In addition, OPN is up-regulated in NSCLC and even more in cells with strong potential and capacity of metastasis and invasion [18, 19]. Wang and colleagues found that OPN was involved in the acquired resistance of lung cancer to afatinib, and its mechanism needs to be further explored [36]. In this study, we found that OPN contributes to acquired resistance by enhancing expression of integrins αV and β3. OPN acts as a ligand for integrins, and OPN/integrin forms a positive feedback pathway to jointly induce acquired resistance.

The ECM alone can induce tumour cell resistance to treatment [37]. As a family of cell surface receptors, integrins play an important role in interaction with the ECM. Integrin biochemical and mechanical signalling regulates cell survival, proliferation, differentiation, migration, adhesion, apoptosis, anoikis, polarity and stemness [38–40]. Integrin αVβ3 is a large family of integrins. Integrin αVβ3 expression and activation drive the intracellular signalling that promotes cancer cell survival, invasion, metastasis, angiogenesis, and self-renewal [39, 41], as well as chemotherapy resistance [42, 43] and radiotherapy resistance [44, 45]. Several studies have reported that integrin β3 is up-regulated after EGFR-TKI treatment [46, 47], consistent with our findings. More importantly, our findings add to current knowledge about how integrin β3 is upregulated in resistant tumours. According to a previous study, the Kras/RalB/NF-κB pathway and miR-483-3p are essential for integrin β3-mediated EGFR-TKI resistance. However, our results showed that up-regulated integrin αVβ3 in gefitinib-resistant cells resulting from OPN down-regulation activated the FAK/Akt/Erk pathway (Fig. 7). Similarly, Kanda and colleagues found that acquired erlotinib resistance was mediated by the integrin β1/Src/Akt signalling pathway in lung cancer [48]. Overexpression of OPN/integrin αvβ3 results in activation of downstream FAK signalling, which is a key component of the signal transduction pathways activated by integrins and has an essential role in cancer cell survival, EMT, metastasis, and stemness [49]. Several studies have shown that activation of FAK signalling is associated with EGFR-TKI resistance [46, 47]. Based on our results, VS-6063 can enhance sensitivity to gefitinib in PC9GR cells. We reason that inhibitors targeting FAK might interact with EGFR TKIs to prevent or delay the occurrence of acquired resistance and progression of lung cancer. However, the interaction between cancer cells and the microenvironment and the mechanisms of EGFR-TKI acquired resistance in tumour cells are rather complicated. Consequently, additional investigations are needed to better understand the roles of the OPN/integrin αvβ3/FAK pathway in acquired resistance to EGFR-TKIs.

There are some limitations to our study. First, the acquired resistance mechanism induced by the OPN/integrin αVβ3 pathway was confirmed only in EGFR-TKI-resistant PC9 cells, and our findings should be validated in various EGFR-TKI-resistant lung cancers. Second, although we demonstrated that OPN/integrin αVβ3 can induce acquired resistance in vitro, we still need to verify this in vivo to fully support our conclusion. Finally, we should investigate how expression of OPN is regulated in the process of acquired resistance, which will be helpful for better understanding and overcoming EGFR-TKI acquired resistance in NSCLC.

## Conclusions

We demonstrate that overexpression of OPN is one of the mechanisms of EGFR-TKI acquired resistance. The mechanism by which OPN confers acquired resistance to EGFR-TKIs may involve integrin αVβ3/FAK pathway activation.

## Supplementary Information

**Additional file 1: Table S1.** Information on siRNA sequences and primer sequences.

**Additional file 2: Fig. S1.** Sensitivity of three EGFR-mutant NSCLC cell lines and drug-resistant cells to gefitinib and osimertinib.

**Additional file 3: Table S2.** The results of specific fold changes in the human soluble receptor array kit ARY012.

**Additional file 4: Fig. S2.** The results of the human soluble receptor array kit ARY012.

**Additional file 5: Fig. S3.** PC9GR cells secreted OPN via Golgi apparatus, secretory autophagy and exosome pathways.

**Additional file 6: Fig. S4.** Expression of mRNA and secretion levels of OPN in osimertinib-resistant cells (PC9GR/OR).

**Additional file 7: Fig. S5.** Correlation of SPP1 expression and ITGAV and ITGB3 in TCGA.

**Additional file 8: Table S3.** Clinical characteristics and gene mutation status of NSCLC patients treated with EGFR-TKI.

**Additional file 9: Table S4.** The results of specific fold changes in the human phospho-kinase array ARY03B.

**Additional file 10: Fig. S6.** The results of human phospho-kinase array ARY03B.

**Additional file 11: Fig. S7.** Under gefitinib treatment, p-FAK in PC9 cells transfected with si-ITGB1 was detected by western blot analysis.

## Data Availability

The datasets used and/or analysed during the current study are available from the corresponding author on reasonable request.

## Abbreviations

EGFR-TKIs: Epidermal growth factor receptor tyrosine kinase inhibitors
NSCLC: Non-small-cell lung cancer
CCK-8: Cell Counting Kit-8
OPN: Osteopontin
GR: Gefitinib resistant
sOPN: Secreted OPN
