# Supplementary material for: Abnormally activated OPN/integrin αVβ3/FAK signalling is responsible for EGFR-TKI resistance in EGFR mutant non-small-cell lung cancer
Source: J Hematol Oncol. 2020 Dec 7;13:169. doi: 10.1186/s13045-020-01009-7 (PMC7720454; doi:10.1186/s13045-020-01009-7)
Supplement: Supplementary file 1 — Additional file 1: Table S1. Information on siRNA sequences and primer sequences. [file 13045_2020_1009_MOESM1_ESM.docx]

**siRNA sequences**

si-FAK-1: 5’-GUAUUGGACCUGCGAGGGA-3’

si-FAK-2: 5’-CGAAUGAUAAGGUGUACGA-3’

si-ITGAV: 5’-GGUCCAAGUUCAUUCAGCAAGGCAA-3’

Si-ITGB3: 5’-CAGAUGUCAUUCCAUAUCATT-3’

Si-ITGB1: 5’-CAGCCCAUUUAGCUACAAATT-3’

Si-OPN-1: 5’-GUCUCACCAUUCUGAUGAATT-3’

Si-OPN-2: 5’-CCGAUGUGAUUGAUAGUCATT-3’

Negative control: 5’-UUCUCCGAACGUGUCACGUTT-3’

**Primer sequences**

| **Nane** | **position** | **Sequences (**5'‑3'**)** |
| --- | --- | --- |
| ITGAV | FP | GGACCATCTCATCACTAAGCGG |
|  | RP | AGCACTGAGCAACTCCACAAC |
| ITGB3 | FP | TGTCCAGCCTAATGACGGG |
|  | RP | TCAGCCCCAAAGAGGGATAA |
| ITGB1 | FP | TGAATGCCAAATGGGACACG |
|  | RP | CAGTGTTGTGGGATTTGCACG |
| OPN | FP | GCCCGCTAGCAAACGCCGACCAAGGAAAAC |
|  | RP | ATTTGCGGCCGCTTAATTGACCTCAGAAGATGCAC |
| GAPDH | FP | TGCACCACCAACTGCTTAGC |
|  | RP | GGCATGGACTGTGGTCATGAG |
