# Supplementary figures and images for: Abnormally activated OPN/integrin αVβ3/FAK signalling is responsible for EGFR-TKI resistance in EGFR mutant non-small-cell lung cancer
Source: J Hematol Oncol. 2020 Dec 7;13:169. doi: 10.1186/s13045-020-01009-7 (PMC7720454; doi:10.1186/s13045-020-01009-7)

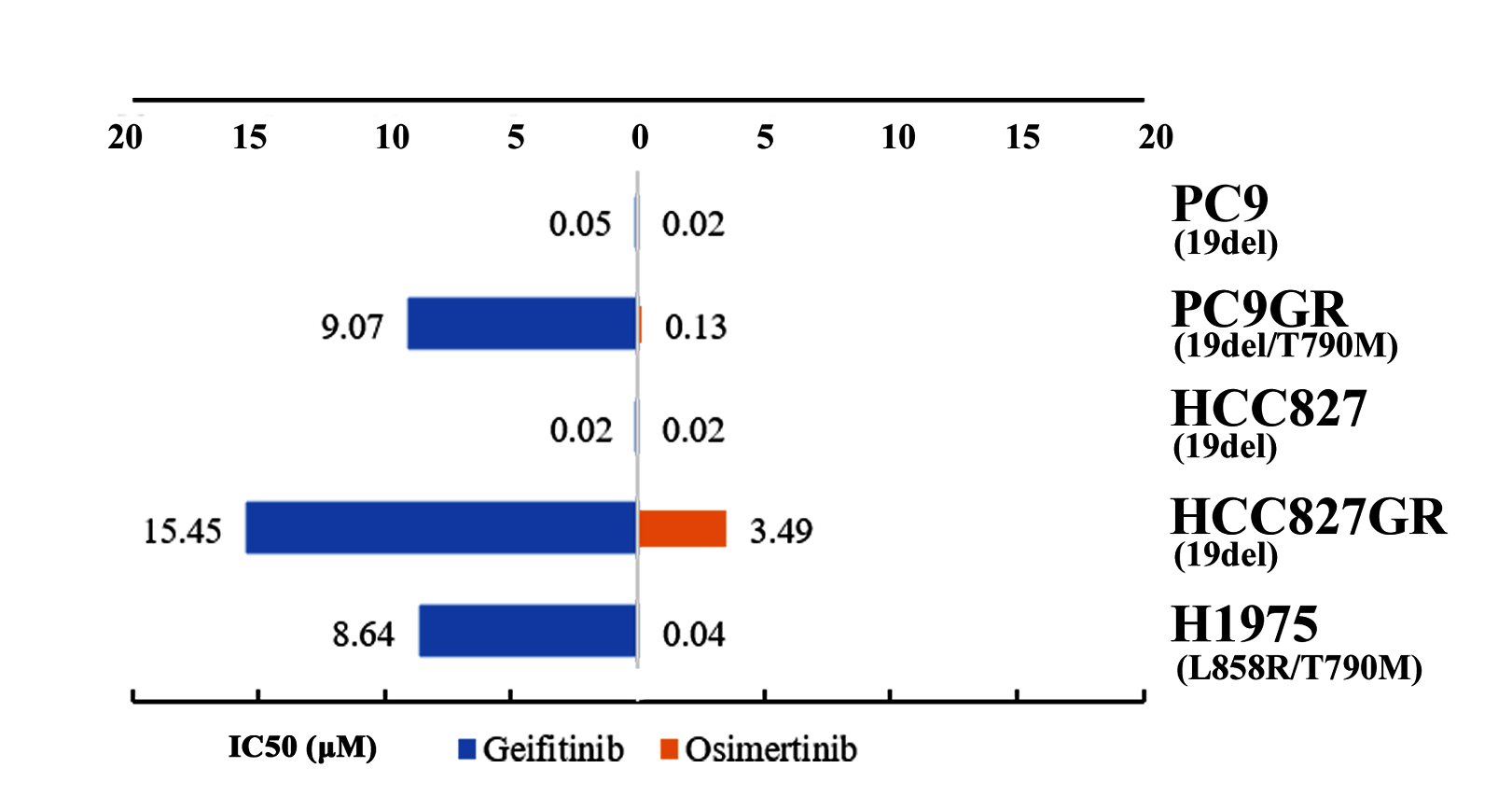

Supplement: Supplementary file 2 — Additional file 2: Fig. S1. Sensitivity of three EGFR-mutant NSCLC cell lines and drug-resistant cells to gefitinib and osimertinib. [file 13045_2020_1009_MOESM2_ESM.tif]

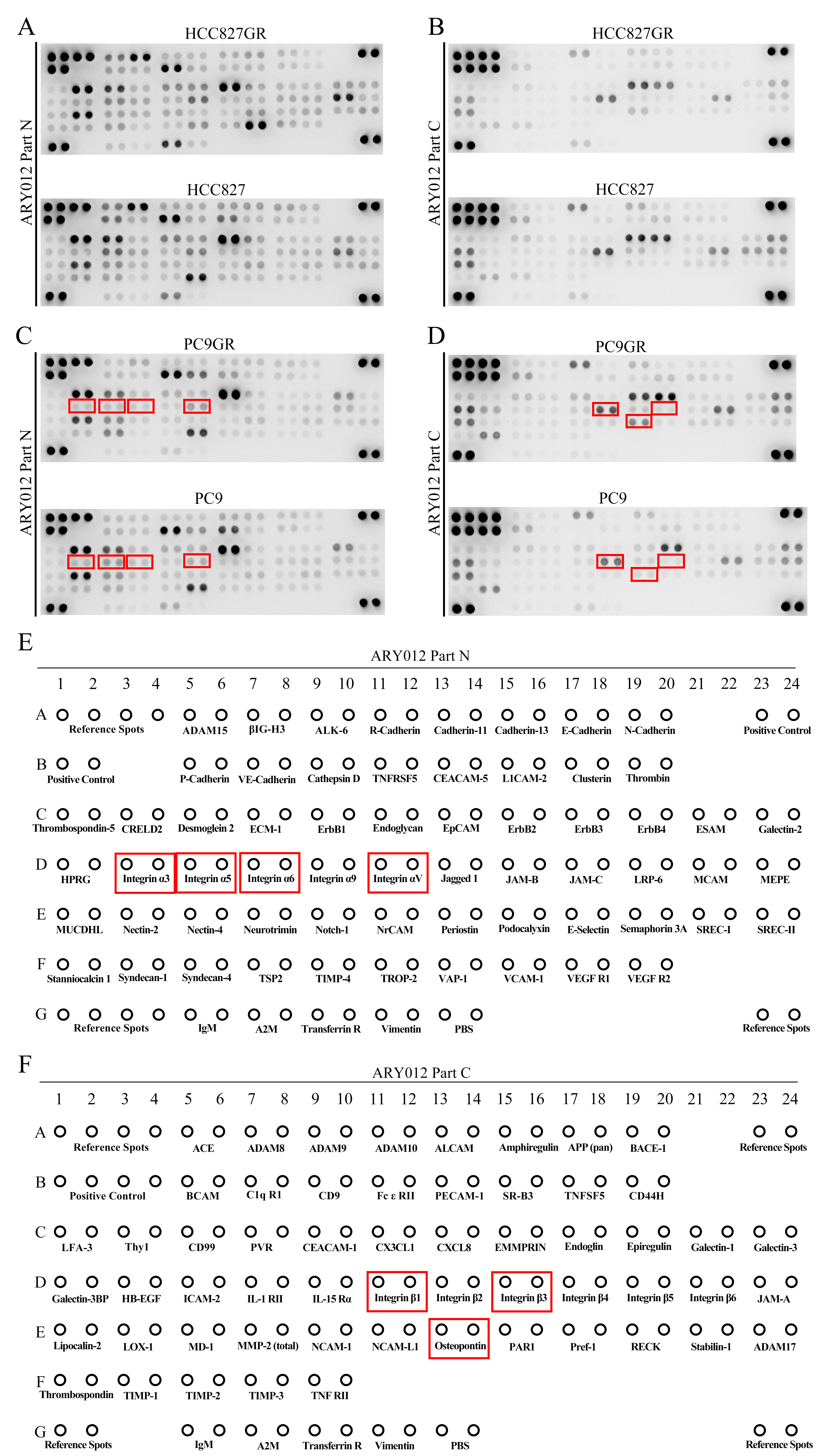

Supplement: Supplementary file 4 — Additional file 4: Fig. S2. The results of the human soluble receptor array kit ARY012. [file 13045_2020_1009_MOESM4_ESM.tif]

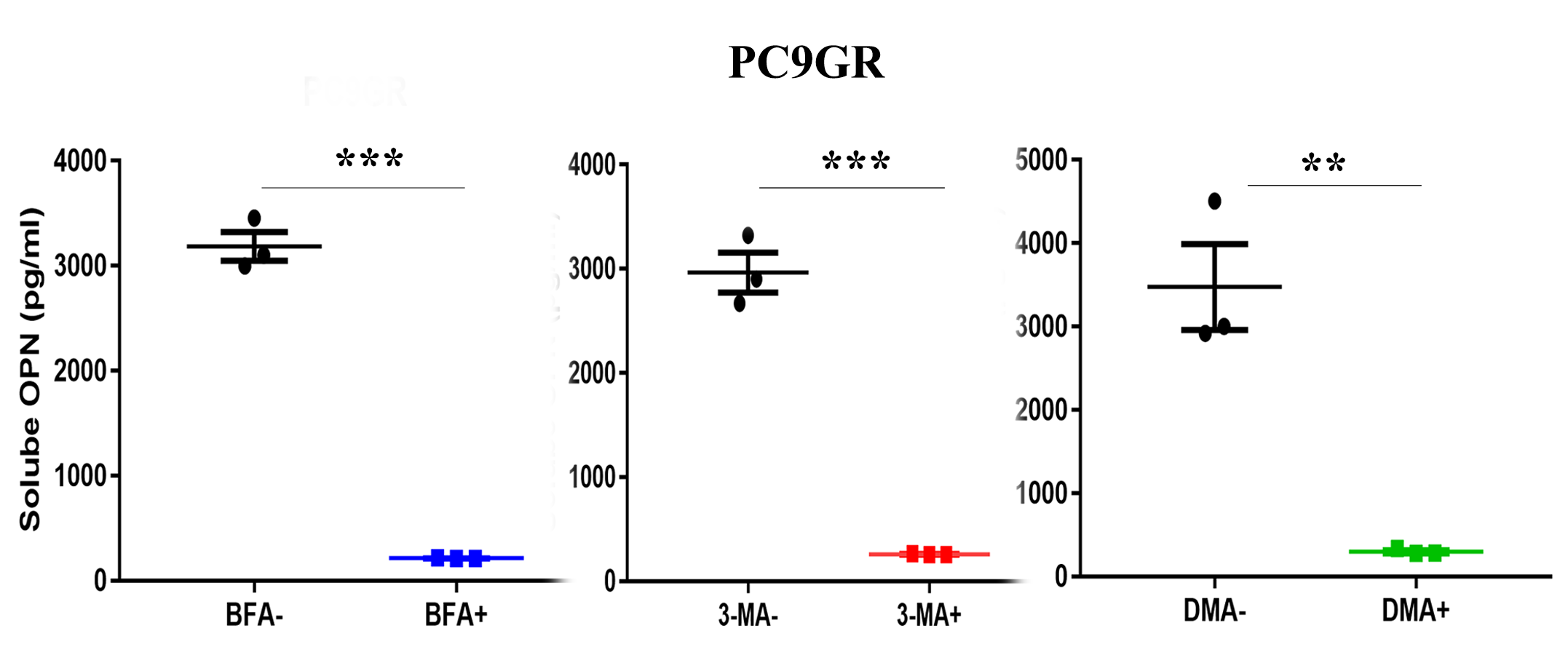

Supplement: Supplementary file 5 — Additional file 5: Fig. S3. PC9GR cells secreted OPN via Golgi apparatus, secretory autophagy and exosome pathways. [file 13045_2020_1009_MOESM5_ESM.tif]

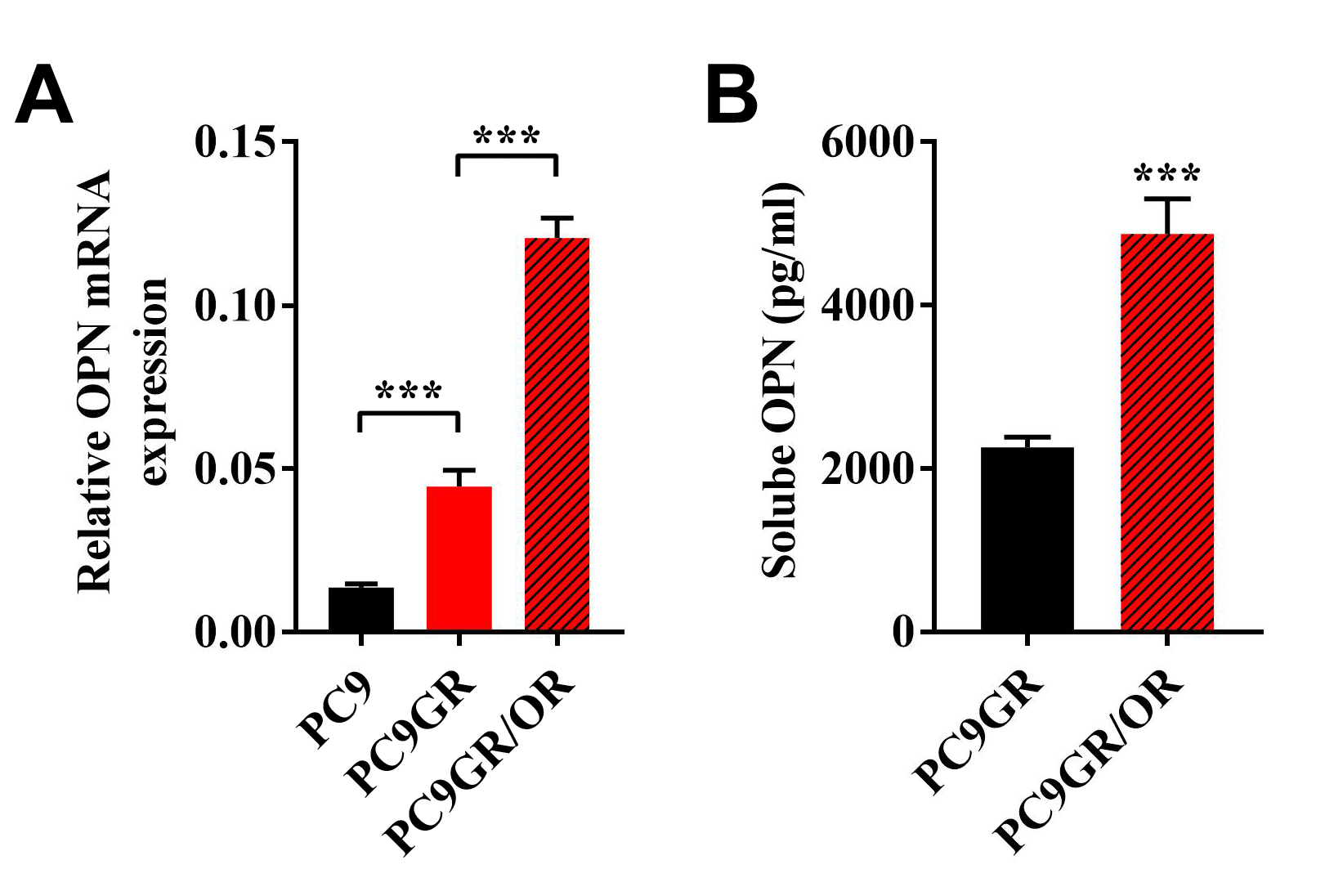

Supplement: Supplementary file 6 — Additional file 6: Fig. S4. Expression of mRNA and secretion levels of OPN in osimertinib-resistant cells (PC9GR/OR). [file 13045_2020_1009_MOESM6_ESM.tif]

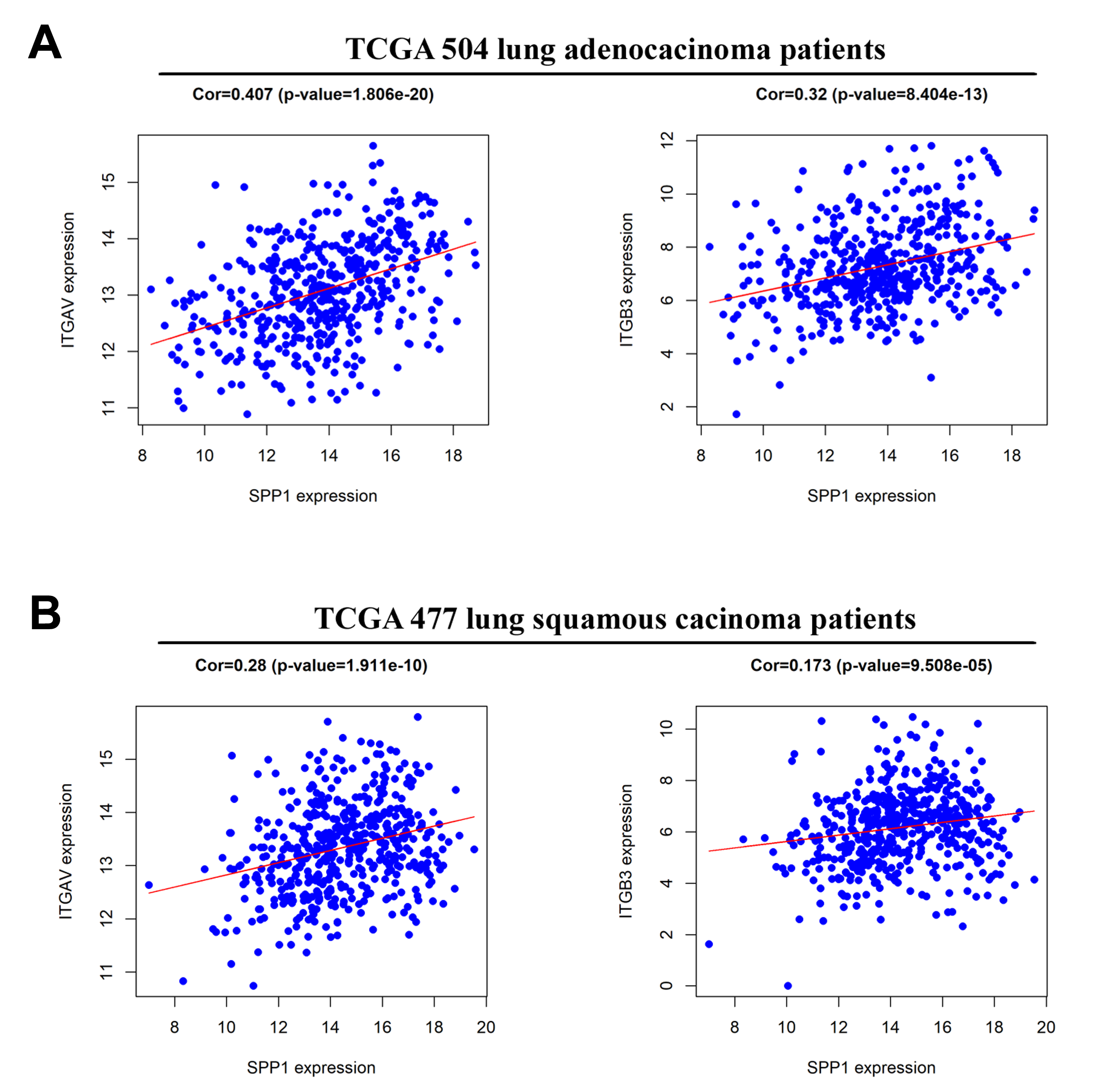

Supplement: Supplementary file 7 — Additional file 7: Fig. S5. Correlation of SPP1 expression and ITGAV and ITGB3 in TCGA. [file 13045_2020_1009_MOESM7_ESM.tif]

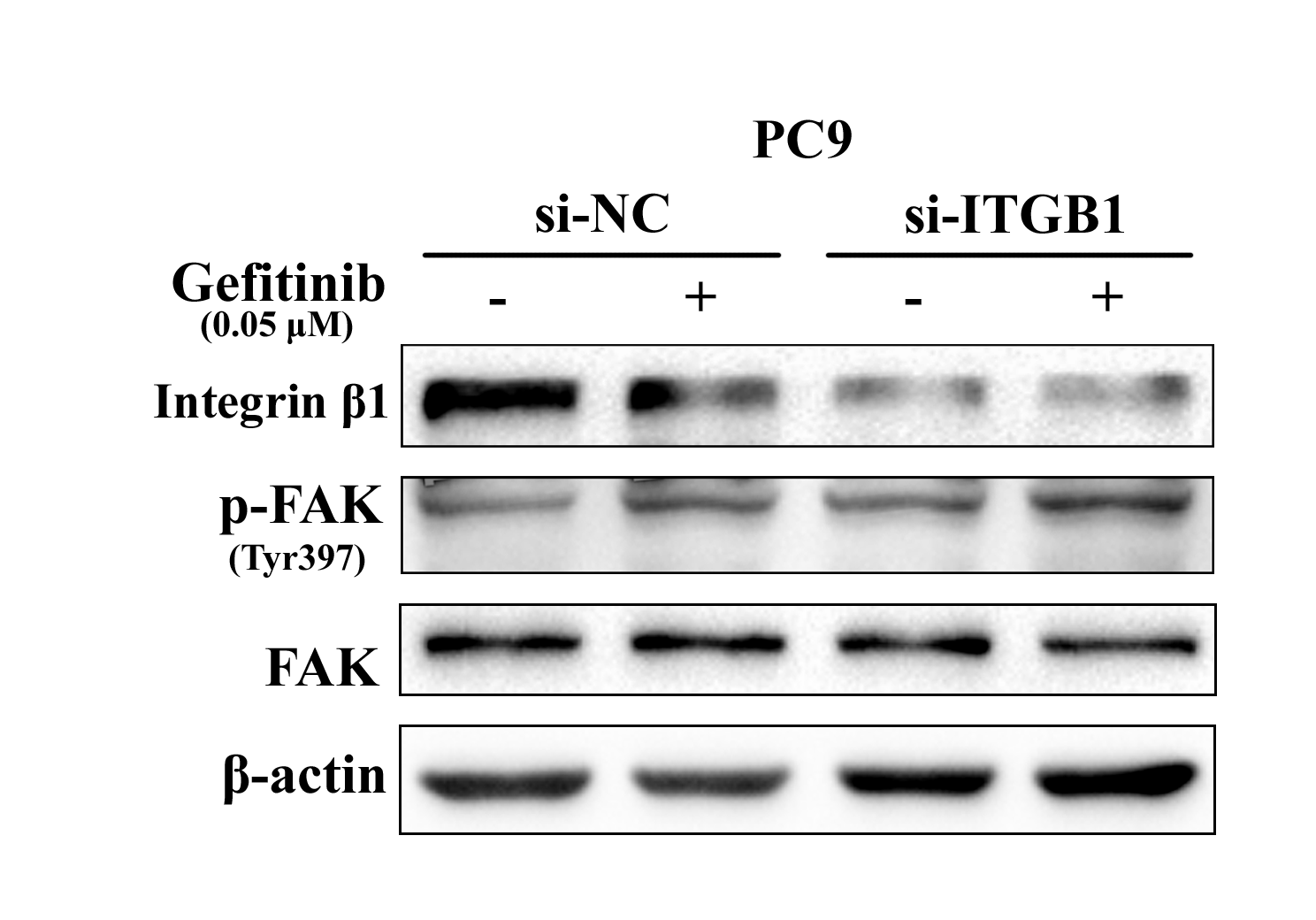

Supplement: Supplementary file 11 — Additional file 11: Fig. S7. Under gefitinib treatment, p-FAK in PC9 cells transfected with si-ITGB1 was detected by western blot analysis. [file 13045_2020_1009_MOESM11_ESM.tif]
