# Supplementary material for: Abnormally activated OPN/integrin αVβ3/FAK signalling is responsible for EGFR-TKI resistance in EGFR mutant non-small-cell lung cancer
Source: J Hematol Oncol. 2020 Dec 7;13:169. doi: 10.1186/s13045-020-01009-7 (PMC7720454; doi:10.1186/s13045-020-01009-7)
Supplement: Supplementary file 3 — Additional file 3: Table S2. The results of specific fold changes in the human soluble receptor array kit ARY012. [file 13045_2020_1009_MOESM3_ESM.docx]

**Supplementary Table 1**. The results of Proteome Profiler Array-Human Soluble Receptor Array Non-hematopoietic panel

| Number | Gene Name | | Fold Change | |
| --- | --- | --- | --- | --- |
|  |  |  | PC-9GR/PC-9 | HCC827GR/827 |
| 1 | CXCL8/IL-8 | | 3.40058966 | 0.915907735 |
| 2 | Osteopontin | | 2.471829703 | 1.50917578 |
| 3 | HB-EGF |  | 1.517779771 | 0.849731829 |
| 4 | CEACAM-1/CD66a | | 1.503952989 | 0.772585082 |
| 5 | Syndecan-4 | | 1.500350097 | 1.088552655 |
| 6 | ADAM9 |  | 1.446411533 | 1.007853248 |
| 7 | CD40/TNFRSF5 | | 1.423113689 | 0.927140308 |
| 8 | CD40 Ligand/TNFSF5 | | 1.334051121 | 0.797568 |
| 9 | EMMPRIN/CD147 | | 1.320737662 | 0.678481 |
| 10 | αIG-H3 |  | 1.30350693 | 0.95093197 |
| 11 | Integrin β5 | | 1.278775514 | 0.754822 |
| 12 | Integrin β2/CD18 | | 1.277488507 | 0.63675 |
| 13 | CD36/SR-B3 | | 1.275944986 | 0.753784 |
| 14 | RECK |  | 1.265883707 | 0.717135 |
| 15 | NCAM-L1 | | 1.251974072 | 0.838734 |
| 16 | CD44H |  | 1.235425877 | 0.833284 |
| 17 | CD31/PECAM-1 | | 1.231076067 | 0.818908455 |
| 18 | CD23/Fc ε RII | | 1.221465759 | 0.838890594 |
| 19 | CD155/PVR | | 1.213880163 | 0.783616153 |
| 20 | MCAM/CD146 | | 1.210664081 | 1.46122127 |
| 21 | Galectin-3BP/MAC-2BP | | 1.204140558 | 0.660899 |
| 22 | Pref-1/DLK-1/FA1 | | 1.199721344 | 0.778169 |
| 23 | IL-15 Rα |  | 1.199273589 | 0.833044 |
| 24 | PAR1 |  | 1.193608599 | 0.936911 |
| 25 | NCAM-1/CD56 | | 1.188170161 | 0.864559 |
| 26 | Integrin β3/CD61 | | 1.188041329 | 0.872874 |
| 27 | APP (pan) |  | 1.183927874 | 0.850994 |
| 28 | Integrin αV/CD51 | | 1.182554565 | 1.176975147 |
| 29 | Galectin-1 |  | 1.1802631 | 0.756301 |
| 30 | CD99 |  | 1.172386226 | 0.765944 |
| 31 | Integrin β1/CD29 | | 1.165570037 | 0.81162 |
| 32 | ADAM10 |  | 1.153846154 | 0.877738 |
| 33 | CX3CL1/Fractalkine | | 1.148828862 | 0.886479 |
| 34 | BCAM |  | 1.145857964 | 0.699639 |
| 35 | C1q R1/CD93 | | 1.14269199 | 0.837339 |
| 36 | Epiregulin |  | 1.140344311 | 0.678481 |
| 37 | BACE-1 |  | 1.138735442 | 0.857519 |
| 38 | Amphiregulin | | 1.136420443 | 0.97959 |
| 39 | ICAM-2/CD102 | | 1.136237015 | 0.793619 |
| 40 | Integrin β4/CD104 | | 1.134907824 | 0.730669 |
| 41 | Galectin-3 |  | 1.130159673 | 0.641337 |
| 42 | IL-1 RII |  | 1.129378404 | 0.858095 |
| 43 | ALCAM/CD166 | | 1.129153522 | 0.666662 |
| 44 | CD9 |  | 1.126100466 | 0.895 |
| 45 | TIMP-1 |  | 1.125222576 | 0.825622 |
| 46 | JAM-A |  | 1.122334346 | 0.572765 |
| 47 | Thrombospondin | | 1.116341849 | 0.899205 |
| 48 | ErbB2/HER2 | | 1.108822736 | 1.030073635 |
| 49 | TIMP-2 |  | 1.107115434 | 0.921898 |
| 50 | ADAM8 |  | 1.105571316 | 0.812586 |
| 51 | MMP-2 (total) | | 1.099771516 | 0.859595 |
| 52 | Endoglin/CD105 | | 1.093101943 | 0.807764 |
| 53 | MD-1 |  | 1.091711912 | 0.867929 |
| 54 | TNF RII/TNFRSF1B | | 1.088179499 | 0.909527073 |
| 55 | BMPR-IB/ALK-6 | | 1.087130899 | 0.855315629 |
| 56 | ACE |  | 1.083855349 | 0.771204 |
| 57 | CD58/LFA-3 | | 1.076697865 | 0.789059474 |
| 58 | Cathepsin D | | 1.076645426 | 0.929807074 |
| 59 | Stabilin-1 |  | 1.072607986 | 0.799096 |
| 60 | TIMP-3 |  | 1.071973603 | 0.918052 |
| 61 | TACE/ADAM17 | | 1.068778539 | 0.821948 |
| 62 | LOX-1/SR-E1 | | 1.062923385 | 0.774838 |
| 63 | SREC-II |  | 1.053321921 | 1.088099952 |
| 64 | EpCAM/TROP-1 | | 1.047458788 | 0.952695054 |
| 65 | Nectin-4 |  | 1.047052479 | 0.837238602 |
| 66 | VCAM-1 |  | 1.033084785 | 4.21658376 |
| 67 | CRELD2 |  | 1.028467292 | 0.983089956 |
| 68 | CD90/Thy1 | | 1.027852862 | 0.842676 |
| 69 | Cadherin-4/R-Cadherin | | 1.007044885 | 0.862879609 |
| 70 | VAP-1/AOC3 | | 0.99385467 | 1.245568163 |
| 71 | TROP-2 |  | 0.991958081 | 0.484398169 |
| 72 | Podocalyxin | | 0.988211375 | 1.106994616 |
| 73 | JAM-B/VE-JAM | | 0.987350778 | 1.005639878 |
| 74 | Lipocalin-2/NGAL | | 0.982748435 | 0.72262 |
| 75 | E-Cadherin | | 0.980506471 | 0.756459481 |
| 76 | SREC-I/SR-F1 | | 0.970541582 | 0.99666159 |
| 77 | N-Cadherin | | 0.969923396 | 0.699786529 |
| 78 | EGFR/ErbB1 | | 0.966003651 | 0.897606566 |
| 79 | Coagulation Factor II/Thrombin | | 0.963762492 | 0.904364824 |
| 80 | Jagged 1 |  | 0.962359164 | 0.937503902 |
| 81 | MEPE |  | 0.961267261 | 1.06827395 |
| 82 | ECM-1 |  | 0.953504204 | 1.008487602 |
| 83 | E-Selectin/CD62e | | 0.953382588 | 1.262151239 |
| 84 | Syndecan-1/CD138 | | 0.950484636 | 0.84655116 |
| 85 | Semaphorin 3A | | 0.949882214 | 1.10168613 |
| 86 | ErbB4/HER4 | | 0.946488217 | 1.140449019 |
| 87 | COMP/Thrombospondin-5 | | 0.943543768 | 0.973178511 |
| 88 | ErbB3/HER3 | | 0.940515767 | 1.148740733 |
| 89 | Galectin-2 |  | 0.940456267 | 1.293484515 |
| 90 | Periostin/OSF-2 | | 0.935980069 | 1.195256376 |
| 91 | VEGF R2/KDR/Flk-1 | | 0.935353969 | 1.18378827 |
| 92 | NrCAM |  | 0.935288277 | 1.024587601 |
| 93 | Clusterin |  | 0.934220786 | 0.881557387 |
| 94 | Integrin α9 | | 0.933606503 | 1.031500906 |
| 95 | CHL-1/L1CAM-2 | | 0.919261917 | 1.006757216 |
| 96 | VE-Cadherin | | 0.910140983 | 0.942272149 |
| 97 | ESAM |  | 0.907762889 | 1.048171569 |
| 98 | Notch-1 |  | 0.906670187 | 1.125485059 |
| 99 | P-Cadherin | | 0.897859041 | 0.681595576 |
| 100 | LRP-6 |  | 0.892267322 | 1.092582271 |
| 101 | Neurotrimin | | 0.885861225 | 1.074474299 |
| 102 | Desmoglein 2 | | 0.878956675 | 0.87129385 |
| 103 | Integrin α3/CD49c | | 0.873172386 | 1.096650527 |
| 104 | VEGF R1/Flt-1 | | 0.871924878 | 1.687813157 |
| 105 | Thrombospondin-2 | | 0.859153488 | 0.933626405 |
| 106 | Cadherin-13 | | 0.851022494 | 0.950460464 |
| 107 | ADAM15 |  | 0.849300562 | 1.007853248 |
| 108 | JAM-C |  | 0.843705753 | 1.165039012 |
| 109 | HPRG |  | 0.837658192 | 1.036482388 |
| 110 | Integrin α6/CD49f | | 0.831769325 | 0.915865039 |
| 111 | MUCDHL |  | 0.830238046 | 1.09173452 |
| 112 | Endoglycan | | 0.820295665 | 1.141468624 |
| 113 | Stanniocalcin 1 | | 0.818896851 | 1.043705388 |
| 114 | Nectin-2/CD112 | | 0.810129188 | 1.234118616 |
| 115 | Integrin β6 | | 0.805239977 | 0.350542 |
| 116 | Cadherin-11 | | 0.794992197 | 0.777818186 |
| 117 | TIMP-4 |  | 0.714273179 | 1.024532505 |
| 118 | Integrin α5/CD49e | | 0.675535266 | 0.807690875 |
| 119 | CEACAM-5/CD66e | | 0.550318129 | 0.49224857 |
