# Supplementary material for: Abnormally activated OPN/integrin αVβ3/FAK signalling is responsible for EGFR-TKI resistance in EGFR mutant non-small-cell lung cancer
Source: J Hematol Oncol. 2020 Dec 7;13:169. doi: 10.1186/s13045-020-01009-7 (PMC7720454; doi:10.1186/s13045-020-01009-7)
Supplement: Supplementary file 8 — Additional file 8: Table S3. Clinical characteristics and gene mutation status of NSCLC patients treated with EGFR-TKI. [file 13045_2020_1009_MOESM8_ESM.docx]

Table 2 Clinical information of patients

patient ID age sex mutation past treatment post mutaion

01 53 female L858R icotinib L858R

02 68 female L858R icotinib L858R

03 53 male L858R erlotinib L858R,MET amp

04 70 female L858R icotinib -----

05 69 female L858R erlotinib L858R,T790M

06 67 female 19del gefitinib 19del,MET amp

07 63 male 19del gefitinib L861G,ERBB2 amp
