# Supplementary material for: Abnormally activated OPN/integrin αVβ3/FAK signalling is responsible for EGFR-TKI resistance in EGFR mutant non-small-cell lung cancer
Source: J Hematol Oncol. 2020 Dec 7;13:169. doi: 10.1186/s13045-020-01009-7 (PMC7720454; doi:10.1186/s13045-020-01009-7)
Supplement: Supplementary file 9 — Additional file 9: Table S4. The results of specific fold changes in the human phospho-kinase array ARY03B. [file 13045_2020_1009_MOESM9_ESM.docx]

**Supplementary Table 3**. The results of Proteome Profiler Array-Human

Phospho-Kinase array

| Number | Gene Name | | Fold Change | |
| --- | --- | --- | --- | --- |
|  |  |  | PC-GR/PC-9 | 827GR/827 |
| 1 | p38α T180/Y182 | | 0.771010994 | 0.681416878 |
| 2 | ERK1/2 T202/Y204, T185/Y187 | | 0.799113236 | 0.564877427 |
| 3 | JNK 1/2/3 T183/Y185, T221/Y223 | | 0.897343558 | 0.605458269 |
| 4 | GSK-3α/β S21/S9 | | 1.004706326 | 0.788272572 |
| 5 | p53 S392 |  | 1.091726066 | 0.804515083 |
| 6 | EGFR Y1086 | | 0.84778025 | 0.715772438 |
| 7 | MSK1/2 S376/S360 | | 0.916762251 | 1.000824197 |
| 8 | AMPKα1 T183 | | 1.121300752 | 0.761577636 |
| 9 | Akt 1/2/3 S473 | | 0.8264608 | 0.747171156 |
| 10 | Akt 1/2/3 T308 | | 0.939077601 | 1.098703653 |
| 11 | p53 S46 |  | 0.985937832 | 0.849978094 |
| 12 | TOR S2448 | | 1.074596862 | 0.770934595 |
| 13 | CREB S133 | | 1.095129117 | 0.787144946 |
| 14 | HSP27 S78/S82 | | 1.061195717 | 0.841748985 |
| 15 | AMPKα2 T172 | | 0.888755085 | 0.888389329 |
| 16 | β-Catenin |  | 0.669726142 | 0.590091839 |
| 17 | p70 S6 Kinase T389 | | 0.910982861 | 0.815584187 |
| 18 | p53 S15 |  | 1.289056595 | 0.74956136 |
| 19 | c-Jun S63 |  | 1.085402137 | 1.058718743 |
| 20 | Src Y419 |  | 1.025879047 | 0.706314081 |
| 21 | Lyn Y397 | | 0.944204356 | 0.846104765 |
| 22 | Lck Y394 |  | 0.865540296 | 0.722711583 |
| 23 | STAT2 Y689 | | 0.892753132 | 0.908563172 |
| 24 | STAT5a Y694 | | 0.842760073 | 0.869751891 |
| 25 | p70 S6 Kinase T421/S424 | | 0.928798409 | 0.854465764 |
| 26 | RSK1/2/3 S380/S386/S377 | | 0.932269885 | 0.925424431 |
| 27 | eNOS S1177 | | 0.982060093 | 0.984799736 |
| 28 | Fyn Y420 |  | 0.832350167 | 0.814238291 |
| 29 | Yes Y426 |  | 1.002166047 | 0.862419825 |
| 30 | Fgr Y412 |  | 0.822710828 | 0.952618094 |
| 31 | STAT6 Y641 | | 0.921486962 | 0.876340763 |
| 32 | STAT5b Y699 | | 0.876948335 | 0.80740101 |
| 33 | STAT3 Y705 | | 0.893784029 | 0.960987625 |
| 34 | p27 T198 |  | 0.995168226 | 1.007991349 |
| 35 | PLC-γ1 Y783 | | 0.807492527 | 0.918121812 |
| 36 | Hck Y411 | | 0.985132136 | 0.789674121 |
| 37 | Chk-2 T68 | | 1.088145044 | 0.810143775 |
| 38 | FAK Y397 | | 1.290749725 | 0.556436355 |
| 39 | PDGF Rβ Y751 | | 0.841468806 | 0.740047576 |
| 40 | STAT5a/b Y694/Y699 | | 0.827987731 | 0.864376379 |
| 41 | STAT3 S727 | | 0.895924397 | 0.684972871 |
| 42 | WNK1 T60 | | 0.962392615 | 0.929673712 |
| 43 | PYK2 Y402 | | 0.852918523 | 0.914251703 |
| 44 | PRAS40 T246 | | 0.968769045 | 1.01275613 |
| 45 | HSP60 |  | 0.850702024 | 0.964528824 |
| 46 | PBS (Negative Control) | |  |  |
| 47 | Reference Spot | |  |  |
